# Elevated soluble thrombomodulin is associated with organ failure and mortality in children with acute respiratory distress syndrome (ARDS): a prospective observational cohort study

**DOI:** 10.1186/s13054-015-1145-9

**Published:** 2015-12-14

**Authors:** Benjamin E. Orwoll, Aaron C. Spicer, Matt S. Zinter, Mustafa F. Alkhouli, Robinder G. Khemani, Heidi R. Flori, John M. Neuhaus, Carolyn S. Calfee, Michael A. Matthay, Anil Sapru

**Affiliations:** Department of Pediatrics, Division of Critical Care, University of California, San Francisco Benioff Children’s Hospital, 550 16th St, Box 0106, San Francisco, CA 94143 USA; Division of Pediatric Critical Care, University of California, San Francisco Benioff Children’s Hospital, 747 52nd St., Oakland, 94609 CA USA; Department of Anesthesia, Critical Care, and Pain Medicine, Massachusetts General Hospital, 55 Fruit St., Boston, 02114 MA USA; Department of Anesthesiology and Critical Care Medicine, Children’s Hospital Los Angeles, 4650 Sunset Blvd., Los Angeles, 90027 CA USA; Department of Epidemiology and Biostatistics, University of California, San Francisco School of Medicine, 550 16th St., San Francisco, 94158 CA USA; Departments of Medicine and Anesthesia, Cardiovascular Research Institute, University of California, San Francisco, 555 Mission Bay Blvd. South, San Francisco, 94158 CA USA

## Abstract

**Introduction:**

The significance of endothelial injury in children with the acute respiratory distress syndrome (ARDS) has not been well studied. Plasma levels of soluble thrombomodulin (sTM), an endothelial surface protein involved in coagulation, have been associated with endothelial injury. We hypothesized that elevated plasma sTM would correlate with mortality and organ failure in children with ARDS.

**Methods:**

We conducted a multicenter prospective observational study of pediatric patients with ARDS between 2008 and 2014. sTM was measured in plasma collected less than 24 hours from ARDS diagnosis. Outcomes were intensive care unit mortality and organ dysfunction by pediatric logistic organ dysfunction scores. Logistic regression was used to adjust for clinically relevant covariates.

**Results:**

Plasma sTM was higher in patients with indirect lung injury compared to direct lung injury (100 ng/mL vs. 86 ng/mL, *p* = 0.02). Increased sTM levels were correlated with more organ dysfunction in the entire study population (Spearman’s rho = 0.37, *p* < 0.01). Overall mortality was 16 %. sTM levels were associated with increased mortality in patients with indirect lung injury (OR 2.7 per log(sTM), *p* = 0.02). These relationships were independent of age, oxygenation defect, or presence of acute kidney injury.

**Conclusion:**

Elevated plasma sTM levels are associated with organ dysfunction in children with ARDS and with higher mortality in children with indirect lung injury. These findings highlight the importance of endothelial injury in children with ARDS and may guide the development of future therapies targeted toward endothelial stabilization, repair, or functional replacement in this population.

**Electronic supplementary material:**

The online version of this article (doi:10.1186/s13054-015-1145-9) contains supplementary material, which is available to authorized users.

## Introduction

Acute respiratory distress syndrome (ARDS) is a disorder historically consisting of hypoxemia accompanied by bilateral pulmonary infiltrates and has been identified as a major contributor to mortality in the pediatric intensive care unit (PICU) population [1, 2]. ARDS involves disruption of the integrity of both the pulmonary endothelium and alveolar epithelium, leading to the development of non-cardiogenic pulmonary edema [3]. While both epithelial and endothelial surfaces are involved in the pathophysiology of the disease, the location of initial insult has classically been used to classify the disease as direct or indirect. Direct lung injury results primarily from injury to the pulmonary epithelium, such as pneumonia or aspiration. In contrast, indirect lung injury develops primarily through damage to the pulmonary endothelium from extrapulmonary sources of systemic inflammation such as sepsis, trauma, or transfusions [4]. Though there is evidence of endothelial injury in patients with ARDS of any etiology, studies in adult patients with ARDS have demonstrated evidence of increased endothelial injury among those with indirect lung injury as compared to those with direct lung injury [5–7]. In addition, endothelial dysfunction is associated with the development of multiple organ dysfunction syndrome (MODS) [8], which is a major mediator of mortality in ARDS [9]. As such, assessment of circulating measures of endothelial injury may improve our understanding of the pathogenesis of ARDS in children, provide a method to risk-stratify children with ARDS, and provide supportive evidence to guide development of future therapeutic interventions.

Thrombomodulin is a transmembrane protein present on all endothelial surfaces and is highly expressed in pulmonary alveolar capillaries [10]. It facilitates the thrombin-mediated conversion of protein C to activated protein C and has roles in coagulation, fibrinolysis, and inflammation [11, 12]. During normal health, thrombomodulin (TM) sheds from the endothelial surface, and soluble thrombomodulin (sTM) circulates at low levels [12]. These circulating fragments have markedly attenuated anticoagulant activity compared with cellular TM or recombinant TM [13, 14]. In the presence of inflammation, neutrophil proteases increase the release of sTM from the cell surface and thereby increase circulating levels of sTM [15]. Studies in children with meningococcal sepsis have documented this effect by demonstrating both depletion of endothelial thrombomodulin and simultaneously increased plasma sTM as compared to controls [16]. sTM levels are similarly elevated in sepsis, disseminated intravascular coagulation (DIC), vasculitis, venous thrombosis, and trauma [17–19]. Experimental and clinical studies have identified sTM as a marker of generalized endothelial injury [20, 21].

sTM has been found to be elevated in the plasma and alveolar edema fluid of adults with ARDS [22], and increased plasma sTM levels are correlated with worse clinical outcomes in adults with ARDS [23–25]. The role of endothelial damage as measured by sTM in children with ARDS and its relative importance in direct versus indirect lung injury are unclear. We hypothesized that sTM levels would be associated with mortality and organ failure in a prospectively studied pediatric population with ARDS and that the association might differ based on the mechanism of lung injury.

## Methods

### Design and patient population

Data were collected between 2008 and 2014 as part of a prospective, multicenter observational cohort of pediatric acute lung injury. Patients were screened for eligibility throughout their stay in five PICUs in California and Wisconsin. Eligibility criteria included acute onset of respiratory symptoms requiring non-invasive or invasive positive pressure support. Patients met the American-European Consensus Conference criteria (referred to in the text as ARDS) [4]. Briefly, eligible patients had an arterial partial pressure of oxygen (PaO2 mmHg) to the fraction of inspired oxygen (FiO2) ratio (P/F ratio) <300 mmHg and had new bilateral infiltrates on chest radiography as judged by site investigators. Patients could also become eligible based on pulse oximetry (SpO2) and an SpO2/FiO2 ratio <253 when PaO2 values were not available [26]. Patients were excluded if they were <30 days of age, <36 weeks corrected gestational age, >18 years of age, had a documented limitation of care order, were wards of the state at the time of screening, or had been enrolled in the cohort previously.

### Ethics, consent and permissions

The institutional review board of the University of California, San Francisco, CA, Oakland Children’s Hospital and Research Center, Oakland, CA, Valley Children’s Hospital, Madera, CA, Children’s Hospital Los Angeles, Los Angeles, CA, and the University of Wisconsin, Madison, WI reviewed and approved the collection of clinical data and biological samples. Informed consent was obtained from patients’ parents or legal guardians.

### Data and sample collection

Demographics, and pre-existing medical conditions, as documented in the medical record, were collected at enrollment. The primary lung injury risk factor was recorded as determined clinically by site investigators. The lung injury group (direct vs. indirect) was then assigned as previously described [4]. After enrollment, daily data including vital signs, respiratory therapies, and laboratory results were recorded. Pediatric risk of mortality (PRISM) III scores were calculated from these data [27]. Vasopressor use was recorded for any dose of one or more of the following: dopamine, epinephrine, norepinephrine, phenylephrine, dobutamine, or milrinone. Patients were followed through hospital discharge. Plasma samples for sTM assays were collected in EDTA tubes within 24 hours of meeting study eligibility criteria. Plasma samples were obtained only from indwelling catheters or during scheduled laboratory collections.

The primary outcome was death prior to intensive care unit (ICU) discharge (ICU mortality). The secondary outcome was the pediatric logistic organ dysfunction (PELOD) score, which is a validated outcome measure of organ dysfunction in critically ill children [28]. The single worst value for each component of the six-organ systems from days 1-7, 14, 21, and 28 after study enrollment were used in the calculation of the PELOD score. The number of failing organ systems was defined as the number of organ systems with a PELOD subscore ≥1 [28].

### Soluble thrombomodulin assay

sTM was measured in plasma samples using two-antibody sandwich enzyme-linked immunosorbent assays (Asserachrom Thrombomodulin assay, Diagnostica Stago, Parsippany, NJ, USA). Samples were assayed in duplicate according to the manufacturer’s protocol and the mean value used for analysis. Samples were used for analysis if the variance between duplicate sTM assays was ≤15 %.

### Statistical analysis

Normally distributed continuous data are reported as mean and standard deviation, and were compared by *t* test. Non-normally distributed, skewed data are reported as median and interquartile range (IQR), and were compared by the Mann–Whitney *U* test. Cuzick’s non-parametric test of trend was used to assess for monotonic trends across ordered groups [29]. Non-normally distributed, skewed variables were log-transformed for use in parametric tests and for graphical representation as indicated. Categorical variables were analyzed by the chi square (χ^2^) test. We used Spearman rank correlation coefficients (rho) to assess and test relationships between variables without assuming normal distributions. We used logistic regression models to quantify the relationships between sTM and mortality and to adjust for covariates. Initially, we assessed the effect of site on the analysis by including site as a random intercept in a mixed effects logistic analysis. The estimated variance of the random intercept was extremely small, consistent with there being no site effect. We therefore used standard logistic regression models for the remainder of the analysis. In view of our sample size we used bivariable models to adjust for one covariate at a time. The Pearson chi square test was used to assess the goodness of fit (GOF) of the data to a logistic model.

We used linear regression analysis to quantify the relationship between sTM and PELOD and to adjust for clinically significant covariates. We adjusted for clinically significant covariates such as age, PRISM III, P/F ratio, and the presence of acute kidney injury (AKI). These covariates were chosen because sTM levels have been reported to vary with age [30], to avoid confounding by the severity of illness and initial lung injury, and AKI was included because sTM is known to be primarily excreted via the kidneys [14]. Due to the small numbers of deaths, the adjusted logistic models for mortality each only included a single potential confounding variable. Patients were deemed to have AKI if one or more of the following criteria were present: urine output <0.5 mL/kg/h, estimated glomerular filtration rate <50 mL/min/1.73 m^2^ body surface area, or receipt of hemodialysis [31]. Receiver operator characteristic curve analysis was used to assess the discrimination of sTM for mortality. Statistical analysis was carried out using Stata 13 (Stata Corporation, College Station, TX, USA).

## Results

### Study population

There were 308 patients enrolled during the study period, of whom 243 had plasma samples available for sTM analysis and formed the study population. There were 65 patients who did not have adequate samples collected for analysis due to absence of indwelling catheters and/or lack of scheduled laboratory collection within the first 24 hours after ARDS diagnosis. Baseline characteristics of the study population are shown in Table 1. Demographic characteristics including age, sex, race/ethnicity, lung injury risk factors, previous medical conditions, and pediatric risk of mortality scores were not statistically different between those patients who did and did not have sTM measurements as shown in Table S1 (see Additional file 1). Mortality for patients with sTM measurements was 16 % compared with 9 % for those without sTM measurements, though this difference was not statistically significant (*p* = 0.17). Patients with sTM measurements had higher rates of vasopressor use and progressed to have higher PELOD scores as shown in Additional file 1, Table S1.

**Table 1:** Baseline characteristics and outcomes for the study population

	All patients (n = 243)	Direct injury (n = 148)	Indirect injury (n = 95)	*P* value^a^
Age, years	6.8 ± 6.0	5.9 ± 5.8	8.3 ± 5.9	<0.01
Male sex, n (%)	136 (56)	87 (59)	49 (52)	0.27
Caucasian, n (%)	152 (62)	92 (62)	60 (63)	0.88
Hispanic/Latino ethnicity, n (%)	92 (38)	62 (42)	30 (32)	0.11
Lung injury risk factor, n (%)				
Pneumonia	135 (56)	135 (92)	0	
Aspiration	9 (4)	9 (6)	0	
Sepsis	56 (23)	0	56 (59)	
Trauma	13 (5)	0	13 (14)	
Multiple transfusions	7 (3)	0	7 (7)	
Other^b^	21 (9)	2 (1)	19 (20)	
Previous medical conditions, n (%)				
None	84 (34)	46 (31)	38 (40)	0.15
Malignancy or bone marrow transplant	39 (16)	26 (18)	13 (14)	0.42
Vasopressor use^c^, n (%)	108 (45)	56 (38)	52 (55)	0.01
PRISM III^d^ raw score, median	12 (6-20)	12 (6-18)	13 (7-21)	0.11
PELOD (IQR)	20 (11, 30)	20 (11, 30)	21 (11, 31)	0.12
Mortality, n (%)	39 (16)	22 (15)	17 (18)	0.53

^a^ *P*-value represents comparison between direct and indirect lung injury groups. ^b^Others include: pancreatitis, leukemia, post-cardiopulmonary bypass, vascular occlusive disease, hepatic failure. ^c^Vasopressor use at any point during the study period. ^d^PRISM III: pediatric risk of mortality

Patients with indirect lung injury were older than patients with direct lung injury (8.3 vs. 5.9 years, *p* <0.01). Indirect lung injury patients also had higher cumulative rates of vasopressor use (55 % vs. 38 %, *p* = 0.01). Other baseline metrics were similar between the two groups (Table 1).

### Mortality and PELOD score

The overall mortality in the study population was 16 %. Mortality in the indirect lung injury group was 18 % compared with 15 % in the direct group, though this difference was not significant (*p* = 0.53). PELOD scores ranged from 0 to 61 (median 20) and were similar between the two lung injury groups (*p* = 0.12) as shown in Table 1.

### sTM levels are associated with mortality only with indirect lung injury

**Fig. 1:**
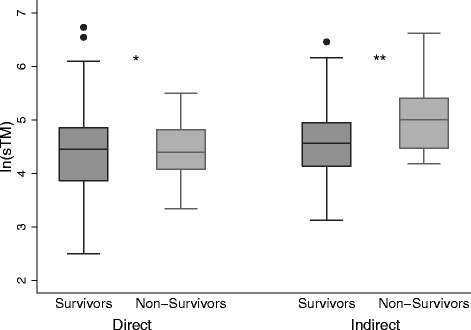
Comparison of soluble thrombomodulin (*sTM*) levels by mechanism of lung injury and mortality. Box plots comparing log-transformed plasma sTM concentrations in survivors (dark gray) (n = 126 for direct lung injury, n = 78 for indirect lung injury) to non-survivors (light gray) (n = 22 for direct lung injury, n = 17 for indirect lung injury), stratified by mechanism of lung injury. sTM concentrations were elevated in non-survivors within the indirect lung injury group but not within the direct lung injury group: *p = 0.9, **p = 0.02

**Fig. 2:**
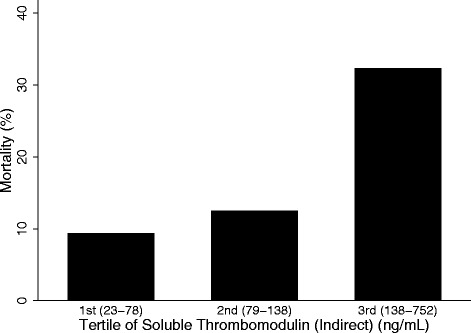
Mortality within the indirect lung injury group (n = 95), stratified by increasing tertiles of soluble thrombomodulin (sTM). sTM ranges for each tertile are shown in parentheses. There is increasing mortality with increasing sTM tertiles (*p* = 0.02 across all tertiles)

The median sTM level among the entire cohort was 93 ng/mL (IQR 57–138). There was no significant difference in sTM levels between survivors (92 ng/mL (IQR 54–135)) and non-survivors (100 ng/mL (IQR 74–160)) (*p* = 0.10). However, upon stratification by the type of lung injury sTM levels were significantly elevated in the indirect lung injury group (100 ng/mL (IQR 68–154)) compared with the direct lung injury group (86 ng/mL (IQR 51–128)) (*p* = 0.02) (Figure S1, Additional file 2). Within the indirect lung injury group sTM levels were significantly elevated among non-survivors (149 ng/mL (IQR 87–223)) compared with survivors (96 ng/mL (IQR 63–142)) (*p* = 0.02) (Fig. 1). This difference was not present within the direct lung injury group (*p* = 0.88). We carried out further analysis of the relationship between sTM and mortality among those with indirect lung injury. On logistic regression analysis within the indirect lung injury group the odds of death were 2.7 times higher for each natural log increase in sTM (95 % CI 1.2–6.1, *p* = 0.02). After performing a goodness-of-fit analysis we did not detect a departure from what would be expected if the data followed a logistic model (*p* = 0.55) (Table 2). This association remained significant after adjustment in bivariable models for age, presence of acute kidney injury or the P/F ratio (Table 2). After adjustment for initial severity of illness by the PRISM III score the independent association between sTM and mortality was no longer present, but the magnitude of the relationship remained similar (odds ratio (OR) 2.4 95 % CI 0.90–6.4, *p* = 0.08). There was a statistically significant interaction between the type of lung injury (direct vs. indirect) and thrombomodulin levels (*p* = 0.06). For receiver operator characteristic analysis of the relationship between sTM and mortality in indirect injury the area under the curve was 0.68 (95 % CI 0.54–0.82) (Figure S2, Additional file 2). Because the relationship between sTM and mortality may be non-linear, we also assessed mortality after stratification of the indirect lung injury group into tertiles (1st: 23–78 ng/mL, 2nd: 79–138 ng/mL, 3rd: 138–752 ng/mL) of sTM levels. In this analysis there was a significant stepwise increase in mortality with increasing sTM tertiles (*p* = 0.02) (Fig. 2).

**Table 2:** Estimated odds ratios assessing the association of soluble thrombomodulin (sTM) levels with mortality in indirect lung injury

	Odds ratio	95 % Confidence interval	*P* value
Log sTM	2.7	1.2-6.1	0.02
Bivariable regression^a^			
Log sTM	2.7	1.2-6.1	0.02
Age, years	1.0	0.91-1.1	0.95
Bivariable regression^b^			
Log sTM	2.5	1.03-6.2	0.04
Acute kidney injury	1.2	0.34-4.2	0.8
Bivariable regression^c^			
Log sTM	2.7	1.1-6.6	0.04
P/F ratio	0.99	0.98-1.0	0.1
Bivariable regression^d^			
Log sTM	2.4	0.9-6.4	0.08
PRISM III raw score	1.0	0.96-1.1	0.5

^a^N = 95, goodness of fit (GOF) *p* value = 0.52. ^b^N = 95, GOF *p* value = 0.52. ^c^N = 89, GOF *p* value = 0.29. ^d^N = 90, GOF *p* value = 0.54. *P/F* ratio of arterial partial pressure of oxygen to fraction of inspired oxygen, *PRISM* pediatric risk of mortality

### sTM levels are associated with organ dysfunction

**Fig. 3:**
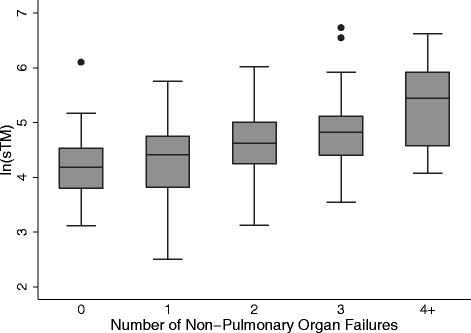
Number of failing non-pulmonary organ systems as a function of soluble thrombomodulin (*sTM*) level. Log-transformed sTM levels among the entire study population stratified by the number of failing non-pulmonary organ systems as assessed by pediatric logistic organ dysfunction: n = 39 for no organ systems, n = 86 for one system, n = 57 for two systems, n = 42 for three systems. Individuals with four or more failing non-pulmonary organ systems are depicted as a single group (n = 19). sTM levels increase with increasing numbers of failing organ systems (*p* <0.01 across all strata)

PELOD scores were then compared to sTM levels. In the entire cohort, there was significant positive correlation between sTM and PELOD (Spearman’s rho = 0.37, *p* <0.01). This correlation was maintained within the direct (rho = 0.33, *p <*0.01) and indirect (rho = 0.42, *p* <0.01) lung injury groups. The association between sTM and PELOD within the entire cohort remained significant using linear regression to model adjustment for age, P/F ratio, and presence of AKI (adjusted coefficient 3.3, 95 % CI 0.65–5.9, *p* = 0.02). PELOD was then stratified by tertile of sTM within the entire cohort. There was a stepwise increase in PELOD score with each increasing tertile of sTM (*p* <0.01) (Figure S3, Additional file 2). This association between elevated sTM levels and PELOD persisted when the cohort was stratified by type of lung injury (Figure S4, Additional file 2). We also tested whether sTM levels were associated with the number of non-pulmonary organ system failures. sTM levels increased when stratified by increasing numbers of failing non-pulmonary organ systems (*p* <0.001) (Fig. 3). This association remained significant upon stratification by mechanism of lung injury (*p* <0.001 for each of direct and indirect lung injury) (Figure S5, Additional file 2). We also tested this association after removal of the renal organ system from the model to account for potential confounding related to sTM clearance changes in AKI, and it remained significant (*p* = 0.002 for direct lung injury and *p* <0.001 for indirect lung injury).

## Discussion

In this study we found that (1) elevated plasma sTM levels within 24 hours of diagnosis in children with ARDS are associated with organ dysfunction, (2) elevated plasma sTM is associated with higher mortality among patients with indirect lung injury, and (3) sTM levels are elevated in indirect lung injury when compared with direct lung injury. These findings emphasize the importance of endothelial injury among pediatric patients with ARDS, especially in patients with an indirect mechanism of lung injury.

The findings are consistent with the need for prolonged respiratory support [24] and increased mortality with elevated plasma sTM among adult patients from the ARDS network studies [25]. The findings are also consistent with studies that reported elevated plasma sTM in pediatric sepsis [16, 32] and associations between plasma sTM and sepsis-related mortality and organ dysfunction in adults [33]. However, the finding in our cohort that organ dysfunction scores also correlate with plasma sTM levels in direct lung injury implies that the relationship is not restricted to septic patients.

Elevated plasma sTM early in the course of the ARDS disease process provides a potential surrogate measurement for the degree of endothelial damage. This is supported by the location of thrombomodulin on the endothelial cell membrane and experimental evidence demonstrating that it is released into the circulation under conditions of inflammation [20]. In addition, plasma sTM levels correlate with the concentration of circulating endothelial cells, which is a metric of endothelial damage [21]. Previous studies of adult patients have also found evidence that endothelial activation and injury are important in the pathogenesis of ARDS. Von willebrand factor (VWF), another potential marker of endothelial dysfunction produced by endothelial cells and platelets, is elevated in patients with non-pulmonary sepsis who go on to develop ARDS [34]. VWF early in the course of ARDS has also been associated with mortality and increased rates of organ failure in adults and a small study in children [35, 36]. In another adult study increased plasma angiopoietin 2, a marker of endothelial dysfunction and vascular permeability, was associated with increased mortality and fewer ventilator-free-days [37]. The outcome of our study is consistent with these findings and helps extend the importance of endothelial injury and dysfunction in ARDS to pediatric populations.

Another possible mechanism for the observed association between elevated plasma sTM and adverse clinical outcomes is through increased intravascular thrombosis, leading to impaired microcirculation and the development of organ dysfunction. Decreased intact cellular thrombomodulin in the setting of inflammation [16] and the observation of relatively decreased anticoagulant activity of its soluble form [13] suggest that patients with elevated plasma sTM are at increased risk of organ injury. Studies of ARDS in animal models and human subjects have suggested that pulmonary coagulopathy plays a significant role in the pathobiology of this disease and its associated comorbidities [38]. Relative deficiencies in proteins such as protein C, antithrombin, tissue factor pathway inhibitor, plasminogen activator inhibitor 1, and thrombomodulin have been implicated in this disruption of the normal balance of procoagulant and anticoagulant forces [38, 39]. The nature of this mechanism suggests that soluble thrombomodulin may be an important mediator of disease in ARDS and an early marker of disease severity.

Despite biological plausibility and promising results in animal models of sepsis and acute lung injury, repletion of natural anticoagulant proteins has thus far shown limited success in human trials [38]. However, none of these trials used biological markers to target these therapies to specific patient populations. Clinical trials have investigated the use of recombinant soluble thrombomodulin therapy in sepsis, DIC, and ARDS [40, 41], though no conclusive pediatric data are available as of yet. These trials did not report pre-treatment sTM levels or use plasma sTM as a basis for targeted therapy with recombinant TM. The results suggest that pediatric patients with ARDS, especially sicker patients with elevated plasma levels of sTM, may be potential candidates for future studies of recombinant thrombomodulin replacement therapy.

The primary strengths of this study are the relatively large size of our pediatric ARDS cohort and the prospective collection of biological samples, which is unusual in this population. The absolute numbers of patients and outcome events also limited our analysis, especially the ability to adjust for multiple potential confounders within a single logistic regression model. We therefore used multiple bivariable models to adjust for one potential confounder at a time. After adjustment for the initial severity of illness by PRISM III the association between sTM and mortality was no longer statistically significant in our logistic model. The magnitude of the association was only slightly attenuated, and the loss of significance may have been related to inadequate sample size. However, our population is reflective of the relatively low frequency of ARDS and decreasing mortality [2] from ARDS in children, and nonetheless we were able to identify significant association between sTM and mortality among patients with indirect lung injury. Another limitation is our inability to obtain plasma samples for analysis of the entire cohort. However, the patients without plasma samples had less organ dysfunction and lower absolute mortality, indicating that those patients were less severely ill and consistent with the fact that they did not require indwelling central lines or arterial lines from which to draw samples. This may limit applicability of our findings to more critically ill ARDS patients.

## Conclusions

In summary elevated soluble thrombomodulin, when measured in children early in the course of ARDS, is associated with increased organ dysfunction and also is associated with increased odds of mortality among children with an indirect mechanism of lung injury. These findings should be validated in additional populations, but they provide evidence of the important role of the magnitude of endothelial injury in determining outcomes from pediatric ARDS. Future interventions targeted toward endothelial stabilization, repair, or functional supplementation may benefit this population.

## Key messages

- sTM, a marker of endothelial injury, is elevated in children with ARDS due to indirect lung injury relative to those with direct lung injury
- sTM levels are independently associated with increased mortality in children with ARDS due to indirect lung injury when measured at the time of ARDS diagnosis
- sTM levels are associated with increasing organ failure scores and number of failing organ systems in children with ARDS

## Additional files

Additional file 1: Table S1. This is a supplementary table providing additional detail about the cohort from the study. Tables are listed here in the order they are referred to in the text. (PDF 85 kb) Additional file 2: Figures S1-S5. This is a collection of supplementary figures to provide additional visual detail about the results of the study. Figures are listed here in the order they are referred to in the text. (PDF 145 kb)

## Abbreviations

AKI: acute kidney injury
Ang-2: angiopoietin 2
ARDS: acute respiratory distress syndrome
CA: California
CI: confidence interval
DIC: disseminated intravascular coagulation
EDTA: ethylenediaminetetraacetic acid
FiO2: fraction of inspired oxygen
GOF: goodness of fit
ICU: intensive care unit
IQR: interquartile range
mmHg: millimeters of mercury
MODS: multiple organ dysfunction syndrome
OR: odds ratio
P/F ratio: ratio of arterial partial pressure of oxygen to fraction of inspired oxygen
PaO2: arterial partial pressure of oxygen
PELOD: pediatric logistic organ dysfunction
PICU: pediatric intensive care unit
PRISM: pediatric risk of mortality
SpO2: oxygen saturation by pulse oximetry
sTM: soluble thrombomodulin
TM: thrombomodulin
VWF: von-Willebrand factor
WI: Wisconsin
