# Supplementary material for: Elevated soluble thrombomodulin is associated with organ failure and mortality in children with acute respiratory distress syndrome (ARDS): a prospective observational cohort study
Source: Crit Care. 2015 Dec 14;19:435. doi: 10.1186/s13054-015-1145-9 (PMC4699329; doi:10.1186/s13054-015-1145-9)
Supplement: Additional file 1: Table S1. — This is a supplementary table providing additional detail about the cohort from the study. Tables are listed here in the order they are referred to in the text. (PDF 85 kb) [file 13054_2015_1145_MOESM1_ESM.pdf]

Title: Elevated soluble thrombomodulin is associated with organ failure and mortality in children with acute respiratory distress syndrome (ARDS): a prospective observational cohort study

Journal Name: Critical Care

Authors: Benjamin E Orwoll*(1, 2); Aaron C Spicer(3); Matt S Zinter(1, 2); Mustafa F Alkhouli (1); Robinder G Khemani (4); Heidi R Flori (2); Carolyn S Calfee (5); Michael A Matthay (5); Anil Sapru (1)

Authors' Institutional Affiliations:

1. Department of Pediatrics, Division of Critical Care, University of California, San Francisco Benioff Children's Hospital, 1975 4th St., San Francisco 94158, CA
2. Division of Pediatric Critical Care, University of California, San Francisco Benioff Children's Hospital, 747 52nd St., Oakland 94609, CA
3. Department of Anesthesia, Critical Care, and Pain Medicine, Massachusetts General Hospital, 55 Fruit St., Boston 02114, MA
4. Department of Anesthesiology and Critical Care Medicine, Children's Hospital Los Angeles, 4650 Sunset Blvd., Los Angeles 90027, CA
5. Departments of Medicine and Anesthesia, Cardiovascular Research Institute, University of California, San Francisco, 555 Mission Bay Blvd. South, San Francisco 94158, CA

Corresponding Author:

Benjamin Orwoll, MD

University of California, San Francisco

orwoll@ucsf.edu

Additional File 1

This is a supplementary table providing additional detail about the cohort from the study.

Tables are listed here in the order they are referred to in the text.

Table S1: Baseline Characteristics and outcomes between those with and without available plasma samples

	Available Plasma: Yes (n=243)	Available Plasma: No (n=65)	*p*-value
Age (y)	6.8 ± 6.0	7.0 ± 6.1	0.87
Male Sex, n (%)	136 (56)	35 (54)	0.76
Caucasian, n (%)	152 (62)	41 (63)	0.94
Hispanic/Latino Ethnicity, n (%)	92 (38)	24 (37)	0.89
Lung Injury Risk Factor, n (%)			0.25
Pneumonia	135 (56)	45 (69)	
Aspiration	9 (4)	1 (2)	
Sepsis	56 (23)	9 (14)	
Trauma	13 (5)	3 (5)	
Multiple Transfusions	7 (3)	0	
Other[a]	21 (9)	7 (11)	
Previous Medical Conditions, n (%)			
None	84 (35)	17 (26)	0.20
Malignancy or Bone Marrow Transplant	39 (16)	8 (12)	0.46
Vasopressor Use[b], n (%)	108 (45)	16 (25)	<0.01
Median PRISM III[c] Raw Score	12 (6-20)	11 (7-14)	0.07
PELOD (IQR)	20 (11, 30)	11 (10, 21)	<0.01
Mortality, n (%)	39 (16)	6 (9)	0.17

[a] Others include: Pancreatitis, Leukemia, post-cardiopulmonary bypass, vascular occlusive disease, hepatic failure

[b] Vasopressor use at any point during the study period

[c] PRISM III: Pediatric Risk of Mortality
