# Supplementary material for: Elevated soluble thrombomodulin is associated with organ failure and mortality in children with acute respiratory distress syndrome (ARDS): a prospective observational cohort study
Source: Crit Care. 2015 Dec 14;19:435. doi: 10.1186/s13054-015-1145-9 (PMC4699329; doi:10.1186/s13054-015-1145-9)
Supplement: Additional file 2: Figures S1-S5. — This is a collection of supplementary figures to provide additional visual detail about the results of the study. Figures are listed here in the order they are referred to in the text. (PDF 145 kb) [file 13054_2015_1145_MOESM2_ESM.pdf]

Title: Elevated soluble thrombomodulin is associated with organ failure and mortality in children with acute respiratory distress syndrome (ARDS): a prospective observational cohort study

Journal Name: Critical Care

Authors: Benjamin E Orwoll*(1, 2); Aaron C Spicer(3); Matt S Zinter(1, 2); Mustafa F Alkhouli (1); Robinder G Khemani (4); Heidi R Flori (2); Carolyn S Calfee (5); Michael A Matthay (5); Anil Sapru (1)

Authors' Institutional Affiliations:

1. Department of Pediatrics, Division of Critical Care, University of California, San Francisco Benioff Children's Hospital, 1975 4th St., San Francisco 94158, CA
2. Division of Pediatric Critical Care, University of California, San Francisco Benioff Children's Hospital, 747 52nd St., Oakland 94609, CA
3. Department of Anesthesia, Critical Care, and Pain Medicine, Massachusetts General Hospital, 55 Fruit St., Boston 02114, MA
4. Department of Anesthesiology and Critical Care Medicine, Children's Hospital Los Angeles, 4650 Sunset Blvd., Los Angeles 90027, CA
5. Departments of Medicine and Anesthesia, Cardiovascular Research Institute, University of California, San Francisco, 555 Mission Bay Blvd. South, San Francisco 94158, CA

Corresponding Author:

Benjamin Orwoll, MD

University of California, San Francisco

orwoll@ucsf.edu

Additional File 2

This is a collection of supplementary figures to provide additional visual detail about the results of the study. Figures are listed here in the order they are referred to in the text.

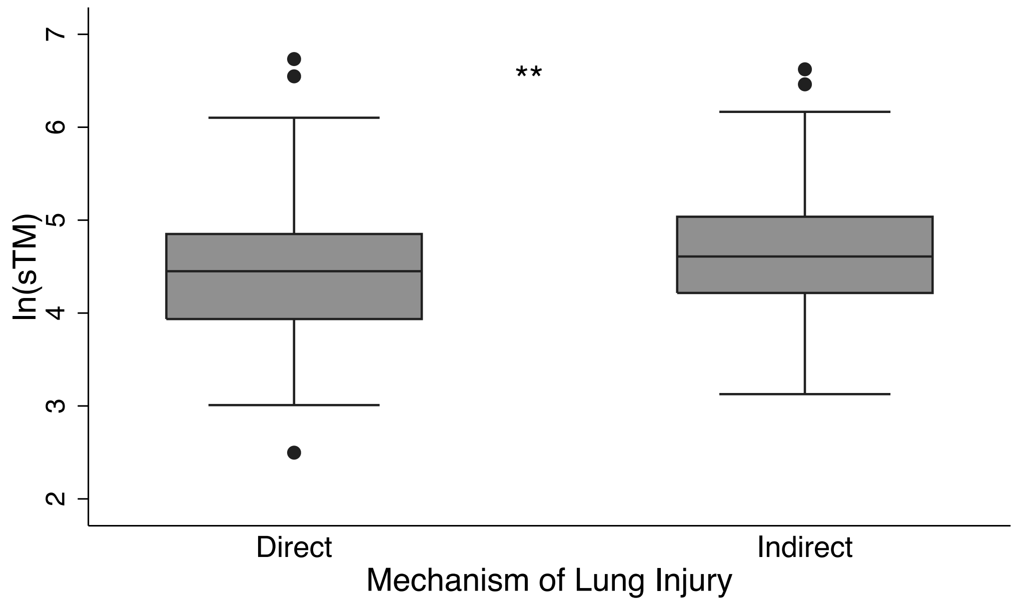

Fig S1 Box plot demonstrating log–transformed sTM levels stratified by mechanism of lung injury. sTM levels were significantly higher in the indirect lung injury group relative to the direct lung injury group.
** p=0.02 for difference between groups

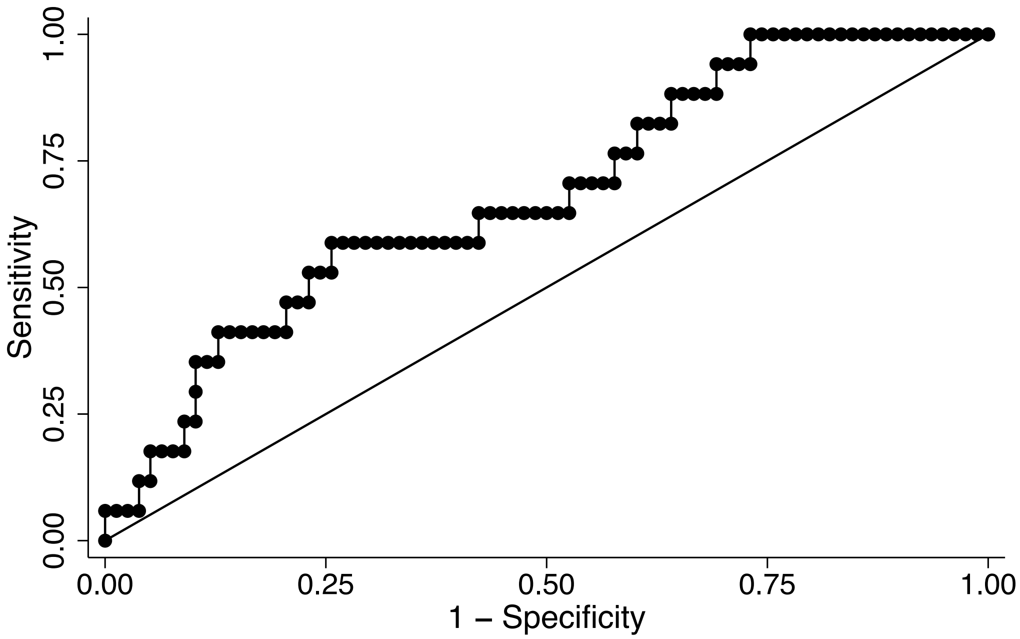

Fig S2 Receiver operator characteristic curve for sTM as a predictor of mortality within the indirect lung injury group. The area under the curve is 0.68 (95% CI 0.54–0.82)

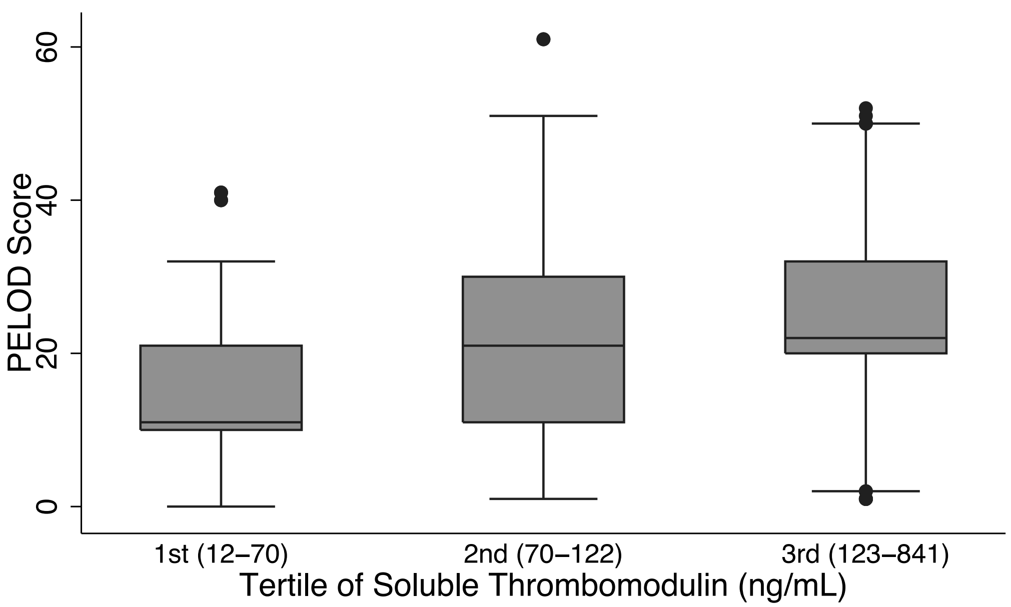

Fig S3 Box plot showing PELOD score as a function of increasing tertiles of sTM among the entire study population. sTM ranges for each tertile are shown in parenthesis. PELOD scores increase with increasing sTM tertiles ($p<0.01$ across all tertiles)

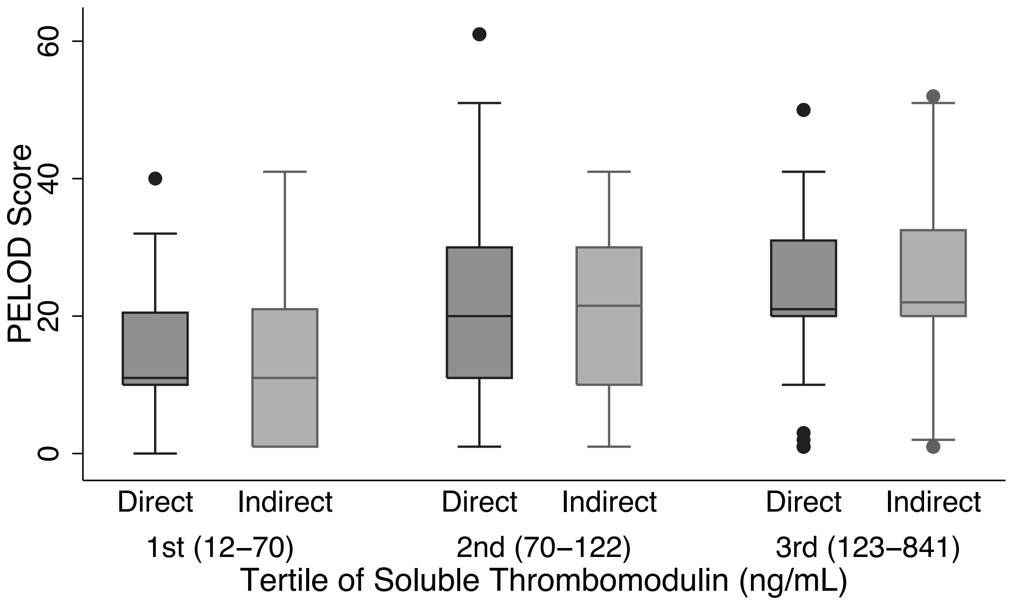

Fig S4 Box plot showing an increasing trend in PELOD score, stratified by mechanism of lung injury, as a function of increasing tertiles of sTM (direct and indirect lung injury in dark grey and light grey, respectively). $p<0.01$ for increase from tertile 1 to tertile 3 within each of the direct and indirect subtypes

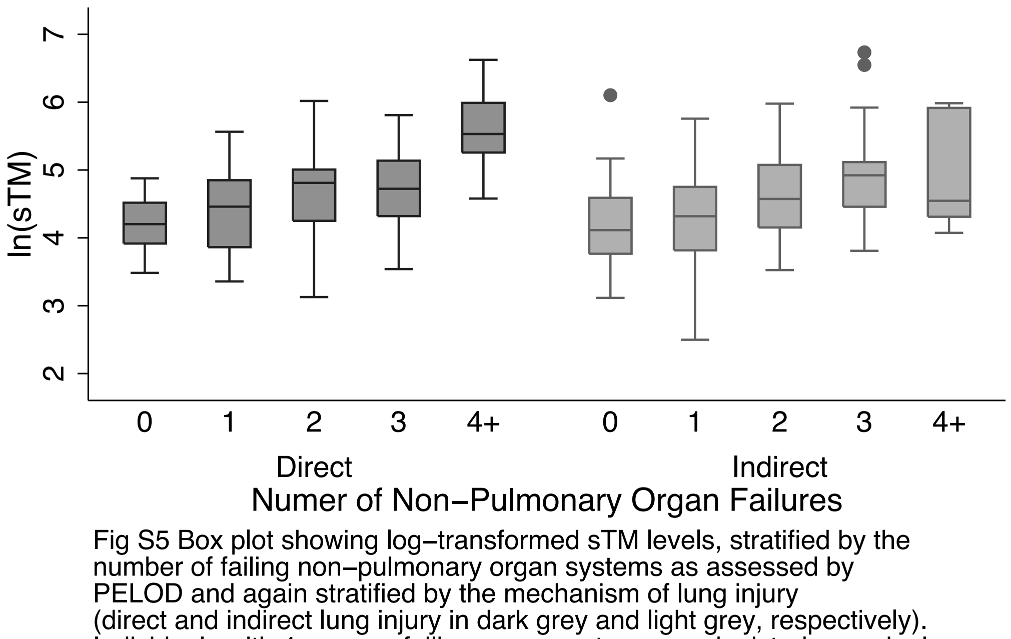

Fig S5 Box plot showing log−transformed sTM levels, stratified by the number of failing non−pulmonary organ systems as assessed by PELOD and again stratified by the mechanism of lung injury (direct and indirect lung injury in dark grey and light grey, respectively). Individuals with 4 or more failing organ systems are depicted as a single group. $p<0.001$ for an increases in sTM levels with increasing organ failures across all strata for each of direct and indirect mechanisms
